# Opioid Use After Elective Otolaryngologic Surgery at a Teaching Institution

**DOI:** 10.31486/toj.21.0054

**Published:** 2022

**Authors:** Basit A. Jawad, Kevin K. Lam, Colleen F. Cecola, Edward D. McCoul

**Affiliations:** ^1^ Department of Otolaryngology–Head and Neck Surgery, Tulane University School of Medicine, New Orleans, LA; ^2^ Department of Otorhinolaryngology and Communication Sciences, Ochsner Clinic Foundation, New Orleans, LA; ^3^ Louisiana State University School of Medicine, New Orleans, LA; ^4^ The University of Queensland Faculty of Medicine, Ochsner Clinical School, New Orleans, LA

**Keywords:** *Analgesics–opioid*, *elective surgical procedures*, *opioid epidemic*, *otolaryngology*, *pain–postoperative*

## Abstract

**Background:** Awareness of the opioid epidemic is promoting opioid stewardship in health care. For many commonly performed procedures in general surgery and gynecology, regimented opioid prescribing practices and/or multimodal nonopioid regimens are adequate for optimizing pain management and minimizing opioid dependence. We investigated opioid prescribing patterns for otolaryngology procedures at a tertiary hospital with the aim of characterizing postoperative pain and opioid use.

**Methods:** This cross-sectional study with a patient survey was conducted in a tertiary care academic otolaryngology practice. Patients ≥18 years who underwent 1 of 41 common surgical procedures at an academic hospital between 2013 and 2017 were enrolled. Patients with any diagnosis of malignancy were excluded. Patients were analyzed according to surgery type (rhinoplasty, sinonasal surgery, tonsillectomy, parotidectomy, thyroidectomy, otologic surgery, and laryngoscopy), and those who had surgery in 2017 were surveyed via telephone interview using a standardized questionnaire.

**Results:** A total of 3,152 patients met the study criteria, of whom 95.7% received an opioid prescription. Commonly prescribed opioid agents were hydrocodone-acetaminophen, oxycodone-acetaminophen, and acetaminophen-codeine. A median of 30 pills was prescribed per surgery, with little variation between different surgery types. Reported patient utilization was highest for parotid surgery and tonsillectomy and lowest for laryngoscopic, thyroid, and otologic surgery. Among all patients who received a prescription for opioids, 5.8% required a refill. Among the surveyed patients, 19.6% reported that they did not obtain the prescribed opioid, while 58.4% said they took half, less than half, or none of the prescribed opioid supply. Only 10.8% of surveyed patients disposed of the excess drugs in a recommended fashion.

**Conclusion:** Our findings showed that the quantity of opioid prescriptions does not reflect actual patient analgesic use for elective surgeries in otolaryngology. Differential analgesic requirements for specific surgeries should be considered when prescribing postoperative analgesia.

## INTRODUCTION

Permissive opioid prescribing practices since the 1990s have resulted in an opioid epidemic in America.^1^ On average, 137 people die daily from opioid-related overdoses in the United States.^1,2^ An estimated 75% of heroin users admit to first abusing a prescribed pain medication.^3^ Increased awareness of the opioid epidemic is functioning as a catalyst to promote opioid stewardship throughout health care. Evidence shows that for many commonly performed procedures in general surgery and gynecology, regimented opioid prescribing practices and/or multimodal nonopioid regimens are adequate for optimizing pain management and minimizing opioid dependence.^2,4^ A burgeoning body of evidence in otolaryngology relates to judicious postoperative analgesia.^5-10^

An estimated 6% of opioid-naive patients undergoing minor or major operations were found to have chronic narcotic use following surgery, regardless of the extent of the surgery.^4^ In 2015, otolaryngologists were responsible for prescribing 922,806 days of opioids to Medicare patients alone.^5^ Even within that cohort, great locoregional variations were identified. Examinations of academic otolaryngology practices reveal disparities between trainees and staff physicians on the appropriate management of postoperative analgesia.^6^ These findings highlight the importance of educational programs on evidence-based regimens for postoperative analgesic management.

Otolaryngologists may benefit from awareness of their prescribing practices to facilitate continuous quality improvement in pursuit of optimal patient care. The aim of this study was to examine opioid prescribing patterns for elective outpatient surgery in otolaryngology and compare this information to patient-reported pain and actual medication use.

## METHODS

This cross-sectional study with a patient survey was approved by the Ochsner Clinic Foundation Institutional Review Board (protocol #2018.227). Patients were included who underwent elective surgery with the Department of Otorhinolaryngology and Communication Sciences at Ochsner Medical Center between January 1, 2013, and December 31, 2017, and were ≥18 years on the date of surgery. Elective surgery was defined as 1 of 41 common outpatient otolaryngology procedures in the Current Procedural Terminology (CPT) code system (Table 1). These CPT codes represent a broad range of surgeries performed in otolaryngology: rhinoplasty, sinonasal surgery, tonsillectomy, parotidectomy, thyroidectomy, otologic surgery, and laryngoscopy (plus interventions). When multiple procedures were performed concurrently, the patient was either grouped into the major type of surgery (ie, surgery involving multiple sinuses and septoplasty was classified as sinonasal) or grouped into the surgery with the greatest perceived pain (ie, multiple-level sleep surgery including tonsillectomy was classified as tonsillectomy). Patients with any diagnosis of malignancy were excluded. Patients were categorized by surgery type, age, and sex and were investigated for prior opioid use.

**Table 1.** Current Procedural Terminology (CPT) Codes of Interest for Each Surgery Type

Surgery Type	CPT Codes
Rhinoplasty	21310 Closed nasal reduction
	21315 Closed nasal reduction
	21320 Closed nasal reduction
	30400 Rhinoplasty, primary
	30410 Rhinoplasty, primary
	30420 Rhinoplasty, primary
	30430 Rhinoplasty, secondary
	30440 Rhinoplasty, secondary, with osteotomies
	30450 Rhinoplasty, secondary, with osteotomies
Sinonasal surgery	31254 Ethmoidectomy, partial (anterior)
	31255 Ethmoidectomy, total
	30130 Excision inferior turbinate
	30140 Submucous resection inferior turbinate
	30520 Septoplasty
	30801 Ablation, soft tissue of inferior turbinate
Tonsillectomy	42821 Tonsillectomy and adenoidectomy
	42826 Tonsillectomy
Parotidectomy	42410 Excision of parotid gland without nerve dissection
	42415 Excision of parotid gland with nerve dissection
Thyroidectomy	60240 Thyroidectomy, total
	60252 Thyroidectomy total with limited neck dissection
	60260 Thyroidectomy, completion
Otologic surgery	69631 Tympanoplasty without mastoidectomy
	69632 with OCR
	69633 with OCR and synthetic prosthesis
	69635 Tympanoplasty with mastoidectomy
	69636 with OCR
	69637 with OCR and synthetic prosthesis
	69641 Tympanoplasty with mastoidectomy, without OCR
	69642 with OCR
	69643 with intact or reconstructed wall, without OCR
	69644 with intact or reconstructed wall, with OCR
	69645 radical or complete, without OCR
	69646 Tympanoplasty: radical or complete, with OCR
	69650 Stapes mobilization
	69660 Stapedectomy
	69661 with footplate drill-out
	69662 Revision stapes surgery
Laryngoscopy	31545 Direct laryngoscopy with removal of TVC lesion
	31570 Direct laryngoscopy with injection augmentation
	31571 Direct laryngoscopy with operating microscope or telescope

OCR, ossicular chain reconstruction; TVC, true vocal cord.

The subset of patients who underwent surgery during calendar year 2017 were further assessed via a telephone interview using a standardized questionnaire to assess analgesic use (Figure). The questionnaire was administered by 3 authors (B.A.J., K.L., C.C.) who contacted each patient using an Ochsner Health research phone line and in accordance with patient privacy. Participants were allowed to skip questions and answer only what they desired. The survey consisted of 6 questions that evaluated patients on perceived postoperative pain 1 week after surgery, prescription compliance, the use of prescribed opioids, refill needs, and disposal method.

**Figure.**
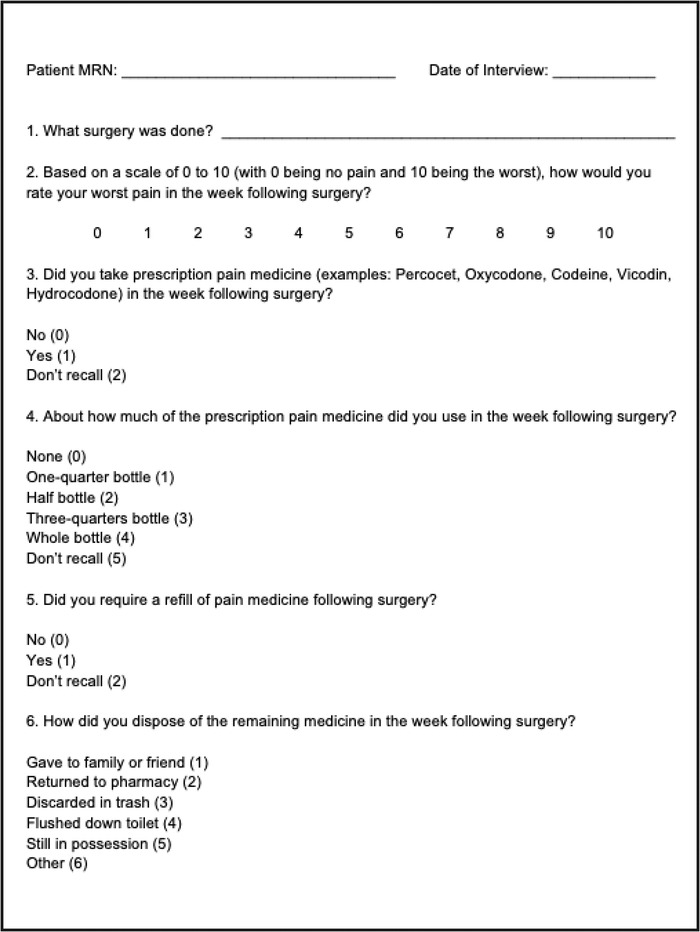
Telephone survey for surgical patients from calendar year 2017.

Postoperative pain was measured on a scale of 0 to 10, with 0 being no pain and 10 being debilitating pain. Prescription compliance was determined by asking if the patient had obtained any of the prescribed pain medicine following surgery. To assess the quantity of pills taken, the amount was stratified based on quartile—none, one-quarter bottle, half bottle, three-quarters bottle, or whole bottle. Patients were asked if they requested a refill of their prescription. Patients were also questioned about how they disposed of the remaining medicine: gave to family or friend, returned to the pharmacy, discarded in trash, flushed down toilet, still in possession, or other. The other category was used to record responses from patients who did not recall how they disposed of the remaining medication or refused to answer.

Data were acquired and analyzed with the assistance of a bioinformatics specialist at our institution. Data were recorded in and retrieved from the Epic electronic medical record (Epic Systems Corp).

## RESULTS

### All Patients Who Received an Opioid Prescription

A total of 3,152 patients met the study criteria during the 60-month study period, of whom 3,017 (95.7%) received a postoperative opioid prescription. The average patient age was 43.2 years with a slight female preponderance (1,726, 57.2%). When stratified by surgery type, the 7 subgroups included the following numbers of patients: 1,357 sinonasal, 701 tonsillectomy, 535 otologic, 261 thyroidectomy, 74 rhinoplasty, 64 laryngoscopy, and 25 parotidectomy. The 3 most commonly prescribed narcotic analgesic medications were hydrocodone-acetaminophen (62.1%), oxycodone-acetaminophen (37.0%), and acetaminophen-codeine (1.0%). Data on postoperative opioid pills prescribed are given in Table 2. A median of 30 pills was prescribed for all surgical procedures, with little variability noted in the quantity of pills prescribed between different types of surgery. Among the patients who received a postoperative opioid prescription, 5.8% were prescribed a refill of the same medication, and 37.6% had a prior (active or inactive) documented opioid prescription in their medical record.

**Table 2.** Postoperative Opioid Prescriptions by Surgery Type, 2013-2017, n=3,017

Surgery Type	Patients, n	Median Pills	Interquartile Range	Hydrocodone, n (%)	Oxycodone, n (%)	Codeine, n (%)	Prior Opioid Use, n (%)
Rhinoplasty	74	24	13	43 (58.1)	31 (41.9)	0 (0)	31 (41.9)
Sinonasal	1,357	30	5	689 (50.8)	655 (48.3)	13 (1.0)	559 (41.2)
Tonsillectomy	701	31	12	602 (85.9)	88 (12.5)	11 (1.6)	246 (35.1)
Parotidectomy	25	30	0	15 (60.0)	10 (40.0)	0 (0)	8 (32.0)
Thyroidectomy	261	31	10	138 (52.9)	123 (47.1)	0 (0)	94 (36.0)
Otologic	535	30	4	338 (63.2)	192 (35.9)	5 (0.9)	168 (31.4)
Laryngoscopy	64	28	10	48 (75.0)	16 (25.0)	0 (0)	27 (42.2)
Total	3,017	30	7	1,873 (62.1)	1,115 (37.0)	29 (1.0)	1,133 (37.6)

### Calendar Year 2017 Subset

Patients who underwent surgery during calendar year 2017 were contacted for a telephone interview. Of 627 patients, 250 (39.9%) agreed to participate and completed the survey. Among these respondents, 49 (19.6%) reported not obtaining the prescribed narcotic provided by their surgeon (Table 3). Refills were reportedly obtained by 23 (9.2%) of surveyed patients. When asked how much of the prescribed opioids they took, 26.0% reported none, 22.0% reported taking the whole bottle, 6.0% reported taking three-quarters of the bottle, 11.6% reported taking half the bottle, 20.8% reported taking one-quarter of the bottle, and 13.6% did not recall how much they took. Reported utilization was highest for parotidectomy and tonsillectomy and lowest for laryngoscopy, thyroid surgery, and otologic surgery (Table 4).

**Table 3.** Telephone Survey Results for Patients Having Surgery During Calendar Year 2017, n=250

Survey Question	n (%)
Prescription filled	
No	49 (19.6)
Yes	194 (77.6)
Don’t recall	7 (2.8)
Amount used from supply	
None	65 (26.0)
One-quarter bottle	52 (20.8)
Half bottle	29 (11.6)
Three-quarters bottle	15 (6.0)
Whole bottle	55 (22.0)
Don’t recall	34 (13.6)
Refill obtained	
No	212 (84.8)
Yes	23 (9.2)
Don’t recall	15 (6.0)
Disposal	
Gave to family/friend	0 (0)
Returned to pharmacy	6 (2.4)
Discarded in trash	43 (17.2)
Flushed down toilet	21 (8.4)
Still in possession	67 (26.8)
Other	113 (45.2)

**Table 4.** Telephone Survey Results for Patients Having Surgery During Calendar Year 2017 by Surgery Type, n=250

Surgery Type	Patients, n	Prescription Not Filled, n (%)	Refill Required, n (%)	Pain Score, Mean (SD)^a^
Rhinoplasty	7	1 (14.3)	0 (0)	4.3 (3.9)
Sinonasal surgery	119	19 (16.0)	8 (6.7)	4.9 (3.1)
Tonsillectomy	42	3 (7.1)	10 (23.8)	6.9 (2.8)
Parotidectomy	3	0 (0)	1 (33.3)	7.3 (1.2)
Thyroidectomy	25	9 (36.0)	0 (0)	3.7 (2.9)
Otologic surgery	43	11 (25.6)	4 (9.3)	5.4 (3.2)
Laryngoscopy	11	6 (54.5)	0 (0)	2.9 (3.6)

^a^The pain scale ranged from 0 to 10, with higher scores indicating greater pain.

In response to the disposal method question, no patient reported giving the excess opioid pills to a friend or family member, whereas 43 (17.2%) patients discarded the excess in the trash and 67 (26.8%) patients said they still had the remaining opioid pills within their possession. Only 27 (10.8%) patients disposed of their opioids in a US Food and Drug Administration (FDA)–recommended method: by returning to a Drug Enforcement Administration–accepted take-back facility (6 patients, 2.4%) or flushing down the toilet (21 patients, 8.4%).^1^ However, 113 (45.2%) patients did not recall how they handled the excess medication or refused to disclose the information.

## DISCUSSION

This study of practice patterns within a tertiary otolaryngology department revealed a frequency of opioid prescription that often exceeded actual patient utilization. More than one-fourth of postoperative survey respondents reported not needing any opioids postoperatively. More than half of interview respondents (58.4%) reported using half or less of the quantity supplied. Patients undergoing rhinoplasty, thyroidectomy, laryngoscopy, and otologic and sinonasal surgery were less likely than tonsillectomy and parotidectomy patients to require opioid analgesia after discharge home. These findings are consistent with similar studies in endocrine surgery, facial plastic surgery, sinus surgery, and laryngology.^8-12^

Although our intention was to introspectively evaluate our opioid prescribing practices and subsequent patient utilization, we aimed to be applicable to a general otolaryngology practice. Our inclusion and exclusion criteria are reflective of a private practice otolaryngologic surgeon. We deliberately selected only common outpatient procedures in otolaryngology. Additionally, major oncologic/reconstructive procedures and patients with a diagnosis of malignancy were excluded, as these subpopulations of patients have special analgesic needs. Our finding that hydrocodone-acetaminophen, oxycodone-acetaminophen, and acetaminophen-codeine were the most common narcotics prescribed in otolaryngology mirrored other institutional studies.^6^

Tonsillectomy and thyroidectomy patients received the greatest quantitative postoperative opioids, while patients undergoing rhinoplasty and direct laryngoscopy (with interventions) received the least quantitative doses. Overall, little variation was noted in the quantity of opioid pills prescribed between different types of surgery. This trend suggests a uniform, albeit inappropriate, approach to analgesic prescribing without regard to the nature of the operation and highlights a potential lack of awareness among surgeons about actual patient needs for postoperative analgesia. This tendency may have resulted from adoption of prescribing practices from other surgical specialties in which more postoperative pain is expected. Alternatively, such practices could arise from susceptibility to marketing efforts from opioid manufacturers or simply from adherence to nonempirical dogma. The otolaryngologist should remain vigilant and accountable for the effects of each of these possible influences.

We found that patients have a considerable knowledge gap regarding best practices for opiate disposal. Only 10.8% of surveyed patients reported disposing of excess narcotics in an FDA-recommended manner. Correct disposal of opioids is essential because inappropriate disposal may potentiate the diversion of the medication for nonmedical use.^1^ Approximately 64% of illicit opioid users report obtaining medications from family or friends, 21% report obtaining medications through a prescription, while only 4% report purchasing opioids through a drug dealer.^13^ These findings illustrate the necessity for physician-driven education about safe medication disposal to decrease the supply of opiates available for diversion.

Although not specifically addressed in our study, multimodal analgesic regimens are available to address perioperative/postoperative patient needs. These techniques include agents with longer durations, regional nerve blocks, gamma-aminobutyric acid agonists, and pain pumps.^14-18^ The Enhanced Recovery After Surgery Society has released evidence-based analgesic recommendations for multiple patient populations, including a proposed guideline for head and neck surgery patients.^14^ The literature has several examples of multimodal regimens for otolaryngology procedures that demonstrate reduced opioid use without increased complication rates.^15-18^

This study has several inherent limitations. Patient selection was limited to 1 regional academic tertiary care institution, and our overall analysis is based on patients’ postoperative course using primarily a single electronic snapshot. The telephone surveys were performed approximately 1 year following each surgical encounter to minimize recall bias, although such bias cannot be removed completely. The fact that 45.2% of survey patients were unable to recall their opioid disposal technique and no survey patients reported family distribution leads to speculation that some patient responses may not have been truthful. Of the patients who agreed to the telephone survey, some surgical categories (ie, parotidectomy and rhinoplasty) had limited participation, reducing the ability to make statistically sound assessments. Further, although we identified the prevalence of prior opioid use, we were unable to stratify these patients to compare them with those who were opioid-naive. Nevertheless, we believe this study is unique in simultaneously evaluating physician opioid prescribing practices and patient-perceived pain and opioid use in otolaryngology.

A multi-institutional randomized controlled trial examining guided pain regimens and patient experience would be valuable to optimize the standardization of postoperative pain management. We found indiscriminate postoperative opioid distribution irrespective to actual patient need in our study. This observation highlights the need to establish educational protocols directed toward the judicious use of postoperative analgesia.

## CONCLUSION

We found a discrepancy between opioid prescriptions and actual patient analgesic use after elective surgeries in otolaryngology. More than 95% of surgical patients received an opioid prescription, with little variation seen in the absolute quantity of pills prescribed. Differential analgesic requirements for specific surgeries should be considered to optimize prescribing practices. These findings demonstrate that education of otolaryngologists and patients about the use of opioids for postoperative analgesia could improve the quality of care and prevent inappropriate diversion of medication.

## Notes

*This article meets the Accreditation Council for Graduate Medical Education and the American Board of Medical Specialties Maintenance of Certification competencies for Patient Care, Medical Knowledge, and Practice-Based Learning and Improvement.*
